# Vital Signs: Leading Causes of Death, Prevalence of Diseases and Risk Factors, and Use of Health Services Among Hispanics in the United States — 2009–2013

**Published:** 2015-05-08

**Authors:** Kenneth Dominguez, Ana Penman-Aguilar, Man-Huei Chang, Ramal Moonesinghe, Ted Castellanos, Alfonso Rodriguez-Lainz, Richard Schieber

**Affiliations:** 1 National Center for HIV/AIDS, Viral Hepatitis, STD, and TB Prevention; 2 Office of the Director, Office of Minority Health and Health Equity; 3 National Center for Injury Prevention and Control; 4 National Center for Emerging and Zoonotic Infectious Diseases; 5 Center for Surveillance, Epidemiology and Laboratory Services, CDC

## Abstract

**Background:**

Hispanics and Latinos (Hispanics) are estimated to represent 17.7% of the U.S. population. Published national health estimates stratified by Hispanic origin and nativity are lacking.

**Methods:**

Four national data sets were analyzed to compare Hispanics overall, non-Hispanic whites (whites), and Hispanic country/region of origin subgroups (Hispanic origin subgroups) for leading causes of death, prevalence of diseases and associated risk factors, and use of health services. Analyses were generally restricted to ages 18–64 years and were further stratified when possible by sex and nativity.

**Results:**

Hispanics were on average nearly 15 years younger than whites; they were more likely to live below the poverty line and not to have completed high school. Hispanics showed a 24% lower all-cause death rate and lower death rates for nine of the 15 leading causes of death, but higher death rates from diabetes (51% higher), chronic liver disease and cirrhosis (48%), essential hypertension and hypertensive renal disease (8%), and homicide (96%) and higher prevalence of diabetes (133%) and obesity (23%) compared with whites. In all, 41.5% of Hispanics lacked health insurance (15.1% of whites), and 15.5% of Hispanics reported delay or nonreceipt of needed medical care because of cost concerns (13.6% of whites). Among Hispanics, self-reported smoking prevalences varied by Hispanic origin and by sex. U.S.-born Hispanics had higher prevalences of obesity, hypertension, smoking, heart disease, and cancer than foreign-born Hispanics: 30% higher, 40%, 72%, 89%, and 93%, respectively.

**Conclusion:**

Hispanics had better health outcomes than whites for most analyzed health factors, despite facing worse socioeconomic barriers, but they had much higher death rates from diabetes, chronic liver disease/cirrhosis, and homicide, and a higher prevalence of obesity. There were substantial differences among Hispanics by origin, nativity, and sex.

**Implications for Public Health:**

Differences by origin, nativity, and sex are important considerations when targeting health programs to specific audiences. Increasing the proportions of Hispanics with health insurance and a medical home (patient-centered, team-based, comprehensive, coordinated health care with enhanced access) is critical. A feasible and systematic data collection strategy is needed to reflect health diversity among Hispanic origin subgroups, including by nativity.

## Introduction

The Hispanic and Latino (Hispanic)* proportion of the population of the United States is projected to increase from 17.7% (56,754,000) in 2015 to 22.8% (84,543,000) by 2035. Hispanics are the largest racial/ethnic minority population in the United States (1). Recent longitudinal data from a seminal study showed important differences in several key health indicators among Hispanics by country or region of origin† subgroups (Hispanic origin subgroups) in four U.S. cities, including prevalence of coronary heart disease, obesity, chronic obstructive pulmonary disease, asthma, and current cigarette smoking (2). In addition, Hispanic life expectancy has been found to be higher for foreign-born Hispanics compared with U.S.-born Hispanics, suggesting that nativity (country of birth) plays an important role in Hispanic health (3). However, published national health estimates stratified by Hispanic origin subgroup and nativity are lacking. The analysis presented in this report used recent mortality and nationally representative health surveillance data to compare death rates for leading causes of death and the prevalences of selected chronic diseases, key risk factors, and health care-related factors among Hispanics, non-Hispanic whites (whites), and Hispanic origin subgroups by nativity and sex to facilitate identification of subpopulations at greatest need of public health interventions.

## Methods

Sociodemographic variables; age-adjusted death rates for the leading causes of death (as ranked for Hispanics overall); prevalences of selected chronic diseases and risk factors; and health insurance status and use of selected health care and preventive services were examined. Analyses were stratified by Hispanics compared with whites, nativity§ (U.S.-born versus foreign-born), and sex. When possible, analyses also were stratified by Hispanic origin subgroups (e.g., Mexicans, Puerto Ricans, and Cubans).

Selected sociodemographic variables and median age were examined using self-reported data from the 2013 American Community Survey (ACS)¶ (all ages, unless otherwise specified) (4).

National Vital Statistics System 2013 mortality data (all ages, 50 states and District of Columbia) were used to determine the leading 15 causes of death for Hispanics and whites, and age-adjusted death rates were calculated for the 15 Hispanic leading causes of death for the following groups: whites, Hispanics, and Hispanic origin subgroups, using methods previously described (5–7). Mortality data for Hispanics from Central America and South America were pooled into a single Central/South American category as some states did when reporting their data to CDC. Death rates for some Hispanic origin subgroups are not reported because of unstable estimates. Corrections were made for both misreporting of race/ethnicity on death certificates and missing data on age. Death rates were adjusted to account for racial/ethnic misclassification using the racial/ethnic-specific and sex-specific classification ratios that CDC derived from the National Longitudinal Mortality Study. Methods for adjustment have been previously described (6).

Data from the National Health Interview Survey (NHIS**) for the period 2009–2013 were used to analyze self-reported disease prevalence for cancer and heart disease, delay and/or nonreceipt of needed health services because of cost, and current cigarette smoking. To examine receipt of recommended cancer screening tests, data were combined from NHIS for 2010 and 2013, the 2 most recent years of available data, and included colorectal tests or procedures, mammograms, and Papanicolaou tests (for women with an intact cervix). Health insurance status was analyzed using NHIS data for the period 2011–2013 (8).

Data from the National Health and Nutrition Examination Survey (NHANES††) for the period 2009–2012 were used to analyze diabetes prevalence (diagnosed and undiagnosed) and the following selected risk factors for heart disease, cancer, and/or diabetes: hypertension, uncontrolled hypertension (among hypertensives), high total cholesterol, and obesity. NHANES data do not include Hispanic origin subgroups other than Mexicans, the only subgroup oversampled.

For NHIS and NHANES data, all variables and age ranges (adults aged 18–64 years except where indicated) are defined in the footnotes of the accompanying tables. The focus on ages 18–64 years, rather than all adults, was driven by the aim to provide data on those adults who could receive the most benefit from early intervention.

In addition, 95% confidence intervals (CIs) were calculated and, in making comparisons, nonoverlapping CIs (a conservative test for statistical significance at alpha = 0.05) were considered indicative of a statistically significant difference. Percentage differences were calculated by dividing the rate or prevalence of interest by the comparison rate (or prevalence), subtracting 1.0, and multiplying by 100%.

## Results

In 2013, Mexicans, Puerto Ricans, and Central Americans together comprised 82.4% of all Hispanics living in the U.S. (64%, 9.5%, and 8.9%, respectively). Hispanics were on average nearly 15 years younger than whites and were twice as likely to live below the poverty line, four times as likely not to have completed high school, and 20 times as likely not to speak English proficiently. (Table 1).

The overall Hispanic all-cause mortality rate was 24% lower than for whites, and Hispanics overall had lower death rates than whites for most leading causes of death (Table 2); notably, this included the two leading causes of death: cancer (−28%) and heart disease (−25%). However, death rates were substantially higher for Hispanics than whites for diabetes (+51%), “chronic liver disease and cirrhosis” (+48%), and homicide (+96%); elevated for “essential hypertension and hypertensive renal disease” (+8%); and similar for “nephritis, nephrotic syndrome, and nephrosis” and “certain conditions originating in the perinatal period.”

Hispanics and whites shared 13 of the 15 leading causes of death (Table 2); homicide and certain conditions originating in the perinatal period were leading causes for Hispanics but not for whites, and “pneumonitis due to solids/liquids” and Parkinson’s disease were leading causes for whites but not for Hispanics. Two out of five deaths (41%) among Hispanics were the result of cancer and cardiovascular disease.

Hispanics had a 49% lower self-reported prevalence of cancer, a 35% lower prevalence of self-reported heart disease, and 133% higher prevalence of diabetes, compared with whites (Table 3). As for risk factors examined, Hispanics less often reported that they smoked, compared with whites (−43%). Hispanics showed a higher prevalence of obesity (+23%) (Table 4) but showed no significant differences for hypertension, uncontrolled hypertension, or high cholesterol. Hispanics were 28% less likely than whites to have had screening tests for colorectal cancer (Table 5). Hispanic women were less likely than whites to have received recommended screening for breast cancer (mammogram) and cervical cancer (Papanicolaou test); these differences were statistically significant but not as pronounced as for colorectal cancer screening (−7% for both) (Table 5). In all, 41.5% of Hispanics lacked health insurance (15.1% of whites), and 15.5% of Hispanics reported delay or nonreceipt of needed medical care because of cost concerns (13.6% of whites) (Table 5).

Stratification by Hispanic origin, sex, and nativity revealed variation in estimates for examined factors (Tables 2–5). For example, self-reported smoking prevalences varied by Hispanic origin as follows: 21.6% (Puerto Ricans), 18.2% (Cubans), 13.0% (Mexicans), and 9.2% (Central/South Americans) (Table 4). The prevalence among Puerto Ricans was similar to that of whites (23.8%) and Cubans. Smoking prevalence among Puerto Ricans was 66% greater than among Mexicans. Smoking prevalence varied significantly among Hispanics by sex: 8.9% among women and 17.7% among men. U.S.-born and foreign-born Hispanics showed significantly different smoking prevalences of 17.7% and 10.3%, respectively.

***Key Points***

- About one in six persons living in the United States are Hispanic or Latino (“Hispanic”). Hispanics on average have lower English proficiency, fewer years of formal education, and higher rates of being uninsured compared with whites.
- Hispanics are not all alike. Country of birth and cultural heritage can make a difference in health behaviors and outcomes.
- Like whites, Hispanics most frequently die from heart disease or cancer. Although Hispanics have lower death rates than whites for nine of the 15 leading causes of death, Hispanic death rates for diabetes and chronic liver disease including cirrhosis are higher by about 50%.
- Ways to improve the health of Hispanics include engaging lay community health workers (“promotores de salud”) to guide persons to needed care by doctors and nurses. Having a medical home, which provides patient-centered, team-based, comprehensive, coordinated health care with enhanced access, is critical. Health education materials need to be written in Spanish and English using culturally appropriate language and situations.
- Additional information is available at http://www.cdc.gov/vitalsigns.

Compared with whites, Mexicans and Puerto Ricans showed 80% greater death rates for diabetes; Mexicans had an 80% greater death rate for chronic liver disease/cirrhosis (Table 2). Puerto Ricans had nearly twice the prevalences of self-reported cancer (+84%) and heart disease (+87%) compared with Mexicans (Table 3). As for differences by sex, although Hispanics overall had hypertension at a prevalence similar to that of whites, hypertensive Hispanic men were 48% more likely than hypertensive Hispanic women to have uncontrolled blood pressure (Table 4). Considering Hispanic origin and sex simultaneously, colorectal cancer screening varied by origin, and women were more likely to be screened (e.g., Cuban men 29%, Cuban women 49%, Puerto Rican men 54%, and Puerto Rican women 61%) (Table 5).

In most instances U.S.-born Hispanics had higher prevalences of risk factors and worse health outcomes than foreign-born Hispanics. U.S.-born Hispanics had a greater prevalence of obesity, hypertension, smoking, heart disease, and cancer than foreign-born Hispanics: 30%, 40%, 72%, 89%, and 93% respectively (Tables 3–4). However, prevalence of high total cholesterol was 45% greater among foreign-born than U.S.-born Hispanics (Table 4). Delay in or not getting medical attention or prescriptions because of cost considerations was similar among foreign-born and U.S.-born Hispanics (Table 5).

## Conclusions and Comment

Compared with whites, Hispanics living in the United States overall had lower death rates for most leading causes of death and lower prevalences of self-reported cancer, heart disease, and current smoking. Hispanics had higher death rates from diabetes, chronic liver disease and cirrhosis, homicide, and essential hypertension and hypertensive renal disease, and they had higher prevalences of obesity and uncontrolled hypertension. They also had decreased access to health care and some preventive care services.

The findings in this report are consistent with previous reports that use the term “Hispanic paradox” (9) to describe Hispanics’ projected longer life expectancy (by an estimated 2 years) (10) and lower overall mortality, despite potential barriers to good health such as higher rates of being uninsured and worse profiles for some social determinants of health. Social determinants of health are conditions “in the environments in which people are born, live, learn, work, play, worship, and age that affect a wide range of health, functioning, and quality-of-life outcomes and risks” (11). Health care has been found to have a substantially lower impact on premature death compared with behavioral factors (12). Lower smoking rates among Hispanics, immigration of healthy immigrants, reverse migration of more ill or elderly immigrants, and higher levels of family support might help to explain this mortality advantage for some Hispanic origin groups (3,9). In addition, being born in the United States and increasing length of time since arrival in the United States are associated with many risk factors and poor health outcomes (13). This also is reflected in the overall poor health status of the United States compared with other developed nations (12).

The present findings, including the similarity of smoking rates among Puerto Ricans and whites (which contrasts with the pattern of lower smoking among Hispanics overall), illustrate the necessity of explicitly considering Hispanic origin subgroup as well as nativity and sex in surveillance and research, including research to better understand the Hispanic paradox and studies of how to intervene to maximize Hispanic health.

Of note, U.S. Hispanics are on average nearly 15 years younger than whites, so early intervention might have a broader impact on Hispanics in preventing chronic diseases that can manifest decades later. Compared with white students, Hispanic students report similar overall tobacco use rates and use of cigarettes and cigars in the past 30 days, and Hispanic middle schoolers report prevalences of 30-day e-cigarette use and hookah use that are two times and four times as high, respectively, as those of white middle school students (14). The health advantages resulting from lower smoking prevalence observed among Hispanics overall might be diminished without timely, culturally, linguistically, and age-appropriate tobacco prevention and cessation interventions for Hispanic youths.

This analysis shows some health disparities affecting Hispanics, including higher diabetes and obesity prevalence and higher death rates related to diabetes and chronic liver disease/cirrhosis compared with whites. In 2013, Hispanics were also shown to have higher proportions than whites of deaths from malignant neoplasms of the liver and intrahepatic bile ducts (1.8% versus 0.8%), and viral hepatitis (0.8% versus 0.3) (15). In both Hispanics and whites, deaths attributed to chronic liver disease and cirrhosis were almost equally divided between alcohol and non-alcohol related (15). Hispanics were recently shown to have a lower prevalence of moderate drinking and a higher prevalence of binge drinking than whites (16). In 2011, Hispanics had higher death rates than whites from chronic hepatitis B virus and hepatitis C virus infections; in 2013, their adult vaccination coverage was similar to whites for hepatitis A virus vaccine but lower for hepatitis B virus vaccine (17). The long-term effects of obesity and diabetes have been associated with chronic liver disease, particularly nonalcoholic fatty liver disease, and liver cancer (18). Liver/intrahepatic bile duct, stomach, and cervical cancers (all associated with infectious etiologies) have been found to be higher among Hispanics compared with whites (19).

Given the presence of multiple Hispanic origin groups residing in the United States, public health programs need to be culturally and linguistically appropriate for Hispanics. Bilingual health education materials, innovative means of increasing health insurance coverage, and access to culturally appropriate health care and preventive services (20) that consider lower health literacy and education levels of many U.S. Hispanics are all critically important. Increasing Spanish-speaking and bilingual health care providers and representation of Hispanics in the health care and public health workforce are focal strategies for improving culturally appropriate and effective health services. Hispanics comprise only 5.8% of U.S. physicians and 7.5% of graduates from schools of public health (21,22).

Hispanics of every age need patient-centered medical homes that provide team-based, comprehensive, coordinated health care with enhanced access. (23). Lay health workers or “promotores de salud” can help provide culturally appropriate health outreach education and screening, linkage to care, and patient navigation (24–26). Examples of CDC-sponsored and other federally-sponsored programs and capacity-building tools for many such programs are available at http://www.cdc.gov/minorityhealth/promotores.html.

This study included data from multiple national data sources. Further, it incorporated data from multiple years to improve stability of estimates for smaller Hispanic subpopulations. This study has several important limitations. Certain variables across all Hispanic origin subgroups could not be assessed because of small sample size. Lower insurance coverage and poorer health care access among Hispanics might have led to underrecognition of disease and consequently, underestimates of self-reported disease prevalence. The quality of Hispanic origin subgroup reporting for mortality data might vary among reporting jurisdictions. Mortality data are subject to racial/ethnic misclassification, but statistical adjustments were made to reduce the potential for bias. Although not a limitation, statistical corrections made for missing ages and racial and ethnic misclassification might limit comparability to reports that do not make these adjustments.

Robust nationwide long-term public health strategies to maximize Hispanic health in the United States need to consider Hispanic origin and nativity. A feasible and systematic data collection strategy is needed to reflect the health diversity in major Hispanic origin subpopulations, including by nativity. Social determinants of health data are important to collect, and oversampling could help ensure representation of relevant Hispanic subpopulations. Studies should be undertaken to better understand what protective factors contribute to Hispanics’ overall lower death rates and to develop Hispanic-focused evidence-based interventions to reduce and eliminate existing health disparities in the areas of diabetes, chronic liver disease/cirrhosis, obesity, and homicide among others.

## Figures and Tables

**TABLE 1:** Selected sociodemographic characteristics of the U.S. population, by nativity, race/ethnicity, and Hispanic/Latino subpopulation — American Community Survey, United States, 2013

Characteristic	Population	% of Hispanic/Latino population	Median age (yrs)	(95% CI)	% with less than a high school diploma*	(95% CI)	% with language other than English spoken at home	(95% CI)	% who speak English less than “very well”	(95% CI)	% living below the poverty line	(95% CI)	% unemployed†	(95% CI)
U.S. population	316,128,839		37.5	(37.4–37.6)	13.4	(13.3–13.5)	20.8	(20.7–20.9)	8.5	(8.4–8.6)	15.8	(15.7–15.9)	5.3	(5.2–5.4)
**U.S.-born** §	274,780,773		35.9	(35.8–36.0)	10.1	(10.0–10.2)	10.7	(10.6–10.8)	1.8	(1.7–1.9)	15.4	(15.3–15.5)	5.4	(5.3–5.5)
**Foreign-born** ¶	41,348,066		43.1	(43.0–43.2)	30.3	(30.1–30.5)	84.0	(83.9–84.1)	49.7	(49.5–49.9)	18.7	(18.6–18.8)	5.0	(4.9–5.1)
**White, non-Hispanic**	197,392,411		42.8	(42.7–42.9)	8.3	(8.2–8.4)	5.4	(5.3–5.5)	1.6	(1.5–1.7)	11.1	(11.0–11.2)	4.3	(4.2–4.4)
**Hispanic/Latino** **	53,986,412	100.0	28.0	(27.9–28.1)	35.3	(35.1–35.5)	73.7	(73.5–73.9)	32.3	(32.1–32.5)	24.8	(24.6–25.0)	6.7	(6.6–6.8)
**Hispanic/Latino subpopulation**														
**Mexican**	34,586,088	64.1	26.2	(26.1–26.3)	40.9	(40.6–41.2)	73.7	(73.4–74.0)	32.3	(32.1–32.5)	26.2	(25.9–26.5)	6.6	(6.5–6.7)
**Puerto Rican**	5,138,109	9.5	28.9	(28.7–29.1)	22.6	(22.1–23.1)	61.9	(61.3–62.5)	17.4	(17.0–17.8)	26.2	(25.6–26.8)	8.0	(7.7–8.3)
**Cuban**	2,013,155	3.7	40.6	(40.4–40.8)	21.0	(20.3–21.7)	79.4	(78.7–80.1)	39.6	(38.8–40.4)	20.0	(19.1–20.9)	6.0	(5.7–6.3)
**Dominican**	1,757,961	3.3	29.0	(28.6–29.4)	31.6	(30.6–32.6)	88.6	(88.1–89.1)	42.2	(41.3–43.1)	28.3	(27.0–29.6)	8.7	(8.2–9.2)
**Central American**	4,802,410	8.9	29.8	(29.6–30.0)	44.9	(44.2–45.6)	87.2	(86.8–87.6)	48.7	(48.2–49.2)	23.3	(22.6–24.0)	6.5	(6.3–6.7)
**South American**	3,260,031	6.0	34.5	(34.2–34.8)	14.9	(14.3–15.5)	83.6	(83.2–84.0)	36.3	(35.7–36.9)	14.9	(14.3–15.5)	5.7	(5.4–6.0)

**Source:** U.S. Census Bureau, American FactFinder, available at http://factfinder.census.gov/faces/nav/jsf/pages/index.xhtml. Based on data from the American Community Survey for the United States, not including Puerto Rico.

**Abbreviation:** CI = confidence interval.

* Among those aged ≥25 years.

† Among those aged ≥16 years.

§ Persons born in the 50 states, District of Columbia, or U.S. territories and includes children born outside the United States to U.S. citizens.

¶ Foreign-born refers to persons born outside the United States or its territories (except for children of U.S. citizens), regardless of current citizenship.

** Persons of Hispanic/Latino ethnicity can be of any race or combination of races.

**TABLE 2:** Leading causes of death* for Hispanics/Latinos and associated death rates† for the U.S. population, non-Hispanic whites, Hispanics/Latinos, and Hispanic/Latino subpopulations — United States, 2013

Leading causes of death (ranked by death counts)§	U.S. population		Race/Ethnicity¶				Hispanic/Latino subpopulation**					
	
			Whites, non-Hispanic		Hispanic/Latino		Mexicans		Puerto Ricans		Cubans	
					
	Mean (per 100,000)	(95% CI)	Mean (per 100,000)	(95% CI)	Mean (per 100,000)	(95% CI)	Mean (per 100,000)	(95% CI)	Mean (per 100,000)	(95% CI)	Mean (per 100,000)	(95% CI)
All causes	736.2	(735.7–736.8)	746.5	(745.9–747.1)	566.6	(564.9–568.2)	588.1	(585.7–590.5)	703.9	(698.1–709.6)	580.5	(575.1–585.9)
1. Malignant neoplasms (2)	166.3	(166.0–166.5)	169.7	(169.4–170.0)	122.2	(121.4–122.9)	123.8	(122.7–124.8)	140.8	(138.3–143.3)	130.7	(128.1–133.3)
2. Diseases of the heart (1)	171.5	(171.2–171.7)	172.7	(172.4 173.0)	128.7	(127.9–129.6)	129.2	(128.1–130.4)	171.5	(168.5–174.4)	153.9	(151.2–156.7)
3. Unintentional injuries (4)	39.3	(39.2–39.4)	43.9	(43.7–44.0)	28.0	(27.6–28.3)	28.7	(28.2–29.1)	32.9	(31.9–34.0)	22.6	(21.5–23.8)
4. Cerebrovascular diseases (5)	37.0	(36.9–37.2)	35.7	(35.6–35.8)	31.7	(31.3–32.1)	35.5	(34.9–36.1)	33.3	(32.0–34.6)	28.3	(27.1–29.4)
5. Diabetes mellitus (7)	21.4	(21.3–21.5)	18.7	(18.6–18.8)	28.3	(27.9–28.6)	33.8	(33.2–34.4)	33.7	(32.4–34.9)	19.6	(18.6–20.6)
6. Chronic liver disease and cirrhosis (12)	10.0	(9.9–10.0)	10.0	(9.9–10.0)	14.8	(14.6–15.1)	18.1	(17.7–18.4)	14.1	(13.4–14.8)	6.5	(5.9–7.1)
7. Chronic lower respiratory diseases (3)	42.0	(41.9–42.1)	46.7	(46.5–46.8)	19.7	(19.4–20.0)	18.3	(17.8–18.7)	26.9	(25.7–28.0)	28.0	(26.8–29.2)
8. Alzheimer’s disease (6)	24.0	(23.9–24.1)	25.3	(25.2–25.4)	18.5	(18.2–18.8)	20.3	(19.8–20.8)	22.2	(21.1–23.4)	19.2	(18.3–20.2)
9. Influenza and pneumonia (8)	15.4	(15.3–15.5)	15.3	(15.2–15.4)	13.6	(13.4–13.9)	14.5	(14.1–14.9)	19.7	(18.7–20.7)	9.5	(8.9–10.2)
10. Nephritis/Nephrotic syndrome and nephrosis (10)	13.3	(13.2–13.3)	12.0	(12.0–12.1)	11.8	(11.5–12.0)	13.5	(13.2–13.9)	13.1	(12.3–13.9)	10.2	(9.5–10.9)
11. Suicide (9)	12.5	(12.4–12.6)	15.6	(15.5–15.7)	6.0	(5.9–6.2)	5.5	(5.3–5.6)	6.9	(6.4–7.3)	8.9	(8.2–9.7)
12. Homicide (–††)	5.3	(5.3–5.4)	2.6	(2.5–2.6)	5.1	(4.9–5.2)	5.2	(5.0–5.3)	6.5	(6.1–6.9)	4.3	(3.7–4.8)
13. Septicemia (11)	10.5	(10.5–10.6)	10.0	(9.9–10.0)	8.7	(8.5–8.9)	9.6	(9.3–9.9)	11.5	(10.8–12.3)	8.0	(7.3–8.6)
14. Certain conditions originating during the perinatal period (–§§)	4.3	(4.2–4.3)	3.4	(3.3–3.4)	3.5	(3.4–3.5)	3.7	(3.6–3.8)	4.6	(4.3–4.9)	2.1	(1.7–2.5)
15. Essential hypertension and hypertensive renal disease (14)	8.3	(8.2–8.4)	7.4	(7.3–7.4)	8.0	(7.8–8.2)	9.2	(8.9–9.5)	8.9	(8.2–9.6)	6.2	(5.6–6.7)

**Source:** Vital Statistic Cooperative Program.

**Abbreviation:** CI = confidence interval.

* Mortality statistics are based on information from all death certificates filed in the 50 states the District of Columbia and provided to the National Center for Health Statistics (NCHS) through the Vital Statistics Cooperative Program. Only causes of death previously defined for ranking purposes by NCHS were ranked (additional information available at http://www.ncbi.nlm.nih.gov/pubmed/24364902). Rankings were based on unadjusted numbers of deaths (not shown in this table) for 2013, not on age-adjusted death rates.

† Age-adjusted rates and 95% confidence intervals were calculated based on average numbers of deaths occurring during 2011–2013. Numbers of persons in the population were based on estimates from the American Community Survey for 2012. The rates were adjusted to account for missing age and racial/ethnic misclassification using the racial/ethnic-specific and sex-specific classification ratios that NCHS derived from the National Longitudinal Mortality Study. Detailed methods for adjustment have been previously described in an NCHS report, available at http://www.cdc.gov/nchs/data/series/sr_02/sr02_148.pdf.

§ Presented in rank order for Hispanics/Latinos, with rank order for all non-Hispanic whites in parentheses for populations overall.

¶ Persons of Hispanic/Latino ethnicity can be of any race or combination of races.

** Because of instability caused by small numbers and the inability to uniquely identify Dominicans, Central Americans, South Americans, and other Hispanics/Latinos in some states, age-adjusted death rates could not be calculated for these Hispanic/Latino subpopulations. Because rates were based on adjusted numbers and were aggregated across the racial/ethnic groups, age-adjusted death rates reported in this analysis might not exactly match age-adjusted death rates calculated by NCHS for this same period.

†† The 13th leading cause of death for non-Hispanic whites (not shown in this table) is Parkinson’s disease.

§§ The 15th leading cause of death for non-Hispanic whites (not shown in this table) is pneumonitis attributable to solids or liquids.

**TABLE 3:** Annualized, age-adjusted prevalence of self-reported cancer, self-reported heart disease, and total diabetes among adults aged 18–64 years, by sex, race/ethnicity, Hispanic/Latino subpopulation, and nativity — United States, National Health and Nutrition Examination Survey (NHANES) 2009–2012,* and National Health Interview Survey (NHIS), 2009–2013†

Characteristic	Population/Group	Cancer§		Heart disease¶		Diabetes**	
		
		Prevalence (%)	(95% CI)	Prevalence (%)	(95% CI)	Prevalence (%)	(95% CI)
**U.S. population**	Overall	3.4	(3.3–3.5)	7.0	(6.8–7.1)	8.1	(6.8–9.6)
	Males	2.2	(2.1–2.4)	7.3	(7.0–7.6)	9.2	(7.2–11.6)
	Females	4.4	(4.3–4.6)	6.6	(6.4–6.9)	7.0	(5.8–8.5)
	U.S.-born††	3.7	(3.5–3.8)	7.6	(7.4–7.8)		
	Foreign-born§§	1.7	(1.5–2.0)	3.7	(3.4–4.0)		
**White, non-Hispanic**	Overall	3.9	(3.7–4.1)	7.5	(7.2–7.7)	6.0	(4.6–7.8)
	Males	2.6	(2.4–2.8)	7.9	(7.6–8.3)	7.3	(5.0–10.5)
	Females	5.2	(4.9–5.4)	7.1	(6.8–7.4)	4.8	(3.4–6.6)
	U.S.-born	3.9	(3.8–4.1)	7.6	(7.3–7.8)		
	Foreign-born	3.0	(2.4–3.9)	5.0	(4.2–6.1)		
**Hispanic/Latino** ¶¶	Overall	2.0	(1.8–2.2)	4.9	(4.6–5.3)	14.0	(11.8–16.5)
	Males	0.9	(0.8–1.2)	4.9	(4.4–5.4)	16.0	(13.5–19.0)
	Females	3.1	(2.7–3.5)	5.0	(4.6–5.5)	12.0	(9.0–15.8)
	U.S.-born	2.7	(2.4–3.1)	6.8	(6.3–7.5)	13.3	(10.1–17.4)
	Foreign-born	1.4	(1.2–1.7)	3.6	(3.2–4.0)	14.0	(11.2–17.5)
**Mexican**	Overall	1.9	(1.6–2.2)	4.7	(4.2–5.1)	15.3	(12.6–18.6)
	Males	0.7	(0.5–0.9)	4.6	(4.0–5.2)	17.9	(14.5–21.9)
	Females	3.2	(2.7–3.7)	4.8	(4.2–5.5)	12.7	(8.8–18.2)
	U.S.-born	2.5	(2.1–3.1)	6.0	(5.4–6.8)	13.3	(9.5–18.4)
	Foreign-born	1.4	(1.1–1.8)	3.6	(3.1–4.2)	16.3	(12.3–21.3)
**Puerto Rican**	Overall	3.5	(2.7–4.5)	8.8	(7.5–10.3)		
	Males	1.9	(1.2–3.2)	9.1	(7.1–11.6)		
	Females	4.9	(3.6–6.6)	8.4	(6.7–10.4)		
	U.S.-born	3.4	(2.6–4.4)	8.9	(7.6–10.5)		
	Foreign-born	—***	—***	—	—		
**Cuban**	Overall	1.5	(0.9–2.5)	4.7	(3.4–6.4)		
	Males	—***	—***	5.3	(3.5–8.0)		
	Females	—***	—***	4.1	(2.5–6.9)		
	U.S.-born	—***	—***	7.2	(4.4–11.6)		
	Foreign-born	—***	—***	3.6	(2.3–5.5)		
**Central American or South American**	Overall	1.4	(1.1–1.9)	3.1	(2.6–3.8)		
	Males	—***	—***	3.4	(2.5–4.7)		
	Females	2.2	(1.6–3.0)	2.9	(2.2–3.8)		
	U.S.-born	—***	—***	—***	—		
	Foreign-born	1.4	(1.0–1.8)	3	(2.4–3.7)		

**Abbreviation:** CI = confidence interval.

* Data from NHIS are age-adjusted to the 2000 U.S. standard population for ages 18–64 years using age groups 18–44, 45–54, and 55–64 years. All estimates are age-adjusted unless otherwise noted. In NHIS, estimates are based on household interviews of a sample of the noninstitutionalized civilian adult population. Unknowns for the columns were not included in the denominators when calculating percentages. Percentages might not add to totals because of rounding. “All adults” includes other races not shown separately.

† Data from NHANES are age-standardized by the direct method to the year 2000 U.S. Census population estimates using age groups 18–24, 25–44, and 45–64 years. In NHANES, estimates are for the noninstitutionalized resident population. “All adults” includes persons of other, non-Hispanic races not shown separately, including non-Hispanic multiracial. Hispanics/Latinos include Mexican-Americans and other Hispanics/Latinos not shown separately.

§ Cancer is based on self-reported responses to questions about whether respondents had ever been told by a doctor or other health professional that they had cancer or a malignancy of any kind. Excludes squamous cell and basal cell carcinomas.

¶ Heart disease is based on responses to questions about whether respondents had ever been told by a doctor or other health professional that they had coronary heart disease, angina (angina pectoris), a heart attack (myocardial infarction), or any other kind of heart disease or heart condition.

** Total diabetes (physician-diagnosed and undiagnosed diabetes). Physician-diagnosed diabetes was obtained by self-report and excludes women who reported having diabetes only during pregnancy. Undiagnosed diabetes is defined as a fasting plasma glucose ≥126 mg/dL or a hemoglobin A1c ≥6.5% and no reported physician diagnosis. Respondents had fasted for ≥8 hours and <24 hours.

†† The definition of “U.S.-born” differs slightly for NHIS and NHANES. In NHIS, “U.S.-born” refers to persons born in the 50 states, District of Columbia, or U.S territories and includes children born outside the United States to U.S citizens. In NHANES, “U.S.-born” refers to persons born in the 50 states or District of Columbia.

§§ The definition of “foreign-born” differs slightly for NHIS and NHANES. In NHIS, “foreign-born” refers to persons born outside the United States or its territories (except for children of U.S. citizens), regardless of current citizenship. In NHANES, “foreign-born” refers to persons born outside the United States, regardless of current citizenship.

¶¶ Persons of Hispanic/Latino ethnicity can be of any race or combination of races.

*** Estimate has a relative standard error >30%.

**TABLE 4:** Prevalence of disease risk factors among adults aged 18–64 years, by sex, race/ethnicity, Hispanic/Latino subpopulation, and nativity — United States, National Health and Nutrition Examination Survey (NHANES) 2009–2012,* and National Health Interview Survey (NHIS), 2009–2013†

Race/Ethnicity and Hispanic subpopulation	Population/Group	Cigarette smoking§		Hypertension¶		Uncontrolled hypertension**		Obesity††		Total high cholesterol§§	
		NHIS (2009–2013)		NHANES (2009–2012)							
		Prevalence (%)	(95% CI)	Prevalence (%)	(95% CI)	Prevalence (%)	(95% CI)	Prevalence (%)	(95% CI)	Prevalence (%)	(95% CI)
**U.S. population**	Overall	21.1	(20.7–21.5)	20.5	(19.4–21.6)	57.7	(52.7–62.5)	34.5	(32.8–36.3)	12.4	(11.4–13.4)
	Males	23.7	(23.1–24.2)	21.8	(20.3–23.4)	65.2	(59.4–70.7)	34.1	(31.9–36.4)	12.0	(10.7–13.2)
	Females	18.5	(18.0–19.0)	19.1	(17.7–20.6)	46.6	(38.0–55.4)	34.9	(32.9–37.0)	12.8	(11.7–13.8)
	U.S.-born¶¶	23.2	(22.8–23.7)								
	Foreign-born***	11.0	(10.4–11.5)								
**White, non-Hispanic**	Overall	23.8	(23.3–24.4)	19.5	(18.1–21.0)	54.4	(47.9–60.7)	32.4	(30.0–34.8)	12.7	(11.4–14.0)
	Male	25.6	(24.9–26.4)	21.1	(18.9–23.4)	61.7	(54.0–68.8)	33.7	(30.9–36.5)	11.8	(10.3–13.3)
	Female	22.0	(21.4–22.7)	17.9	(16.0–19.9)	46.9	(39.6–54.3)	31.1	(27.7–34.5)	13.6	(12.1–15.0)
	U.S.- born	24.1	(23.6–24.7)								
	Foreign-born	17.4	(15.7–19.4)								
**Hispanic** †††	Overall	13.5	(12.9–14.0)	16.8	(15.1–18.6)	67.7	(60.0–74.7)	39.9	(37.1–42.6)	13.3	(11.4–15.2)
	Male	17.7	(16.9–18.6)	17.5	(15.1–20.3)	74.7	(65.8–82.0)	37.7	(34.5–40.9)	15.1	(12.4–17.8)
	Female	8.9	(8.3–9.6)	15.9	(14.1–18.0)	50.5	(36.9–64.0)	41.9	(38.7–45.1)	11.6	(9.5–13.7)
	U.S.-born	17.7	(16.8–18.7)	20.9	(18.2–23.8)	62.6	(50.3–73.4)	47.1	(43.5–50.6)	10.0	(8.5–11.5)
	Foreign-born	10.3	(9.6–11.0)	14.9	(13.0–17.0)	65.9	(56.0–74.6)	36.3	(33.3–39.2)	14.5	(11.9–17.1)
**Mexican**	Overall	13.0	(12.3–13.6)	17.5	(15.6–19.6)	72.4	(62.5–80.4)	42.4	(39.6–45.1)	12.1	(9.9–14.2)
	Male	17.5	(16.4–18.6)	17.2	(14.7–19.9)	79.4	(67.7–87.7)	39.2	(35.5–43.0)	13.7	(10.7–16.7)
	Female	8.0	(7.3–8.7)	17.8	(15.3–20.7)	56.8	(39.3–72.7)	45.7	(41.8–49.5)	10.4	(8.0–12.8)
	U.S.-born	16.0	(15.0–17.2)	21.7	(18.3–25.7)	65.5	(49.7–78.4)	46.8	(42.7–50.8)	9.5	(7.6–11.4)
	Foreign-born	10.6	(9.7–11.5)	14.9	(12.7–17.4)	74.7	(65.8–81.9)	40.0	(37.0–42.9)	13.3	(10.4–16.2)
**Puerto Rican**	Overall	21.6	(19.4–24.0)								
	Male	26.4	(22.8–30.4)								
	Female	17.4	(15.1–19.9)								
	U.S.- born	21.9	(19.7–24.3)								
	Foreign-born	—§§§	—§§§								
**Cuban**	Overall	18.2	(15.3–21.5)								
	Male	22.0	(17.7–27.0)								
	Female	13.6	(10.5–17.5)								
	U.S.-born	21.0	(15.6–27.6)								
	Foreign-born	16.2	(13.1–20.0)								
**Central American or South American**	Overall	9.2	(8.1–10.4)								
	Male	12.5	(10.8–14.3)								
	Female	5.7	(4.5–7.1)								
	U.S.-born	11.7	(8.8–15.4)								
	Foreign-born	9.0	(7.8–10.3)								

**Abbreviation:** CI = confidence interval.

* Data from NHIS are age-adjusted to the 2000 U.S. standard population for ages 18–64 years using age groups 18–44, 45–54, and 55–64 years. All estimates are age-adjusted unless otherwise noted. In NHIS, estimates are based on household interviews of a sample of the noninstitutionalized civilian adult population. Unknowns for the columns were not included in the denominators when calculating percentages. Percentages might not add to totals because of rounding. “All adults” includes other races not shown separately.

† Data from NHANES are age-standardized by the direct method to the year 2000 U.S. Census population estimates using age groups 18–24, 25–44, and 45–64 years. In NHANES, estimates are for the noninstitutionalized resident population. “All adults” includes persons of other, non-Hispanic races not shown separately, including non-Hispanic multiracial. Hispanics/Latinos include Mexican-Americans and other Hispanics/Latinos not shown separately.

§ Current cigarette smoking is based on two survey questions. All respondents were first asked, “Have you smoked at least 100 cigarettes in your entire life?” Respondents answering “yes” were then asked, “Do you now smoke cigarettes every day, some days, or not at all?” Current smokers have smoked at least 100 cigarettes in their lifetime and currently smoke every day or some days.

¶ Hypertension was defined as systolic blood pressure ≥140 mmHg or diastolic blood pressure ≥90 mmHg or currently taking medication to lower blood pressure.

** Uncontrolled hypertension was defined as systolic blood pressure ≥140 mmHg or diastolic blood pressure ≥90 mmHg among those with hypertension.

†† Obesity is defined as body mass index (BMI) ≥30.0 kg/m^2^. BMI was calculated as weight in kilograms divided by height in meters squared (kg/m^2^) rounded to the nearest tenth. Pregnant females excluded from analysis.

§§ High total cholesterol is defined as total cholesterol ≥240mg/dL.

¶¶ The definition of “U.S.-born” differs slightly for NHIS and NHANES. In NHIS, “U.S.-born” refers to persons born in the 50 states, District of Columbia, or U.S territories and includes children born outside the United States to U.S citizens. In NHANES, “U.S.-born” refers to persons born in the 50 states or District of Columbia.

*** The definition of “foreign-born” differs slightly for NHIS and NHANES. In NHIS, “foreign-born” refers to persons born outside the United States or its territories (except for children of U.S. citizens), regardless of current citizenship. In NHANES, “foreign-born” refers to persons born outside the United States, regardless of current citizenship.

††† Persons of Hispanic/Latino ethnicity can be of any race or combination of races.

§§§ Estimate has a relative standard error >30%.

**TABLE 5:** Annualized prevalence of lack of health insurance, nonutilization of medical care or prescription drugs, and use of preventive screening tests for cancer among adults, by sex, race/ethnicity, Hispanic/Latino subpopulation, and nativity — United States, National Health Interview Survey (NHIS), 2011–2013, 2009–2013, or 2010 and 2013*

Race/Ethnicity and Hispanic/Latino subpopulation	Population/Group	Uninsured† (18–64 yrs, 2011–2013)		Delay or nonreceipt of needed medical care during the past 12 months because of cost§ (age-adjusted¶) (18–64 yrs, 2009–2013)		Nonreceipt of needed prescription drugs in the past 12 months because of cost** (age-adjusted) (18–64 yrs, 2009–2013)		Use of colorectal tests or procedures (crude)†† (18–64 yrs, 2009–2013)		Use of mammography in the past 2 years among women (crude)§§ (50–74 yrs, 2010–2013)		Use of Pap tests in the past 3 years in women¶¶ (crude) (21–65 yrs, 2010 and 2013)	
		Prevalence (%)	(95% CI)	Prevalence (%)	(95% CI)	Prevalence (%)	(95% CI)	Prevalence (%)	(95% CI)	Prevalence (%)	(95% CI)	Prevalence (%)	(95% CI)
**U.S. population**	Overall	20.8	(20.4–21.3)	13.9	(13.6–14.1)	10.2	(9.9–10.5)	58.7	(57.8–59.6)				
	Males	23.2	(22.6–23.7)	12.8	(12.5–13.1)	8.3	(7.9–8.6)	57.7	(56.3–59.0)				
	Females	18.6	(18.2–19.1)	14.9	(14.6–15.2)	12.1	(11.8–12.5)	59.6	(58.4–60.9)	72.5	(71.4–73.6)	81.7	(81.0–82.4)
	U.S.-born***	17.3	(16.9–17.7)	14.1	(13.8–14.3)	10.4	(10.1–10.8)	60.1	(59.2–61.1)	73.0	(71.8–74.2)	83.6	(82.8–84.3)
	Foreign-born†††	37.7	(36.6–38.9)	13.2	(12.7–13.6)	9.3	(8.8–9.8)	45.4	(43.2–47.7)	69.5	(66.8–72.0)	73.5	(71.7–75.3)
**White, non-Hispanic**	Overall	15.1	(14.6–15.5)	13.6	(13.3–13.9)	9.5	(9.1–9.9)	60.8	(59.8–61.9)				
	Male	16.5	(16.0–17.1)	12.5	(12.2–12.9)	7.6	(7.2–8.1)	60.0	(58.5–61.5)				
	Female	13.6	(13.2–14.1)	14.6	(14.2–15.0)	11.3	(10.9–11.8)	61.6	(60.1–63.0)	73.3	(72.0–74.6)	83.5	(82.6–84.3)
	U.S.-born	14.9	(14.4–15.3)	13.7	(13.3–14.0)	9.6	(9.3–10.0)	61.0	(59.9–62.0)	73.3	(71.9–74.6)	83.9	(83.0–84.8)
	Foreign-born	19.3	(17.6–21.1)	12.3	(11.3–13.4)	6.8	(5.6–8.1)	57.5	(52.3–62.6)	74.0	(67.8–79.3)	75.0	(69.6–79.8)
**Hispanic/Latino** §§§	Overall	41.5	(40.4–42.6)	15.5	(15.1–15.9)	12.5	(11.9–13.1)	43.7	(41.4–46.1)				
	Male	45.3	(44.2–46.5)	14.5	(14.0–15.0)	10.4	(9.7–11.2)	39.4	(35.8–43.1)				
	Female	37.4	(36.3–38.6)	16.5	(16.0–17.0)	14.7	(13.8–15.6)	47.8	(44.7–50.8)	67.9	(64.9–70.8)	77.7	(76.0–79.3)
	U.S.-born	25.9	(25.1–26.8)	14.8	(14.3–15.4)	12.8	(11.9–13.8)	53.0	(49.4–56.7)	70.5	(66.0–74.7)	81.6	(79.4–83.7)
	Foreign-born	54.7	(53.3–56.1)	16.0	(15.5–16.6)	12.2	(11.5–13.0)	36.5	(33.5–39.6)	66.0	(61.9–69.8)	74.4	(72.0–76.7)
**Mexican**	Overall	45.6	(44.2–46.9)	15.3	(14.8–15.9)	12.8	(12.0–13.6)	41.6	(38.4–44.9)				
	Male	48.8	(47.3–50.2)	14.4	(13.8–15.0)	10.6	(9.6–11.7)	36.8	(32.9–41.8)				
	Female	42.1	(40.7–43.6)	16.4	(15.8–17.0)	15.2	(14.0–16.4)	46.3	(42.4–50.4)	66.8	(62.4–71.0)	76.6	(74.4–78.6)
	U.S.-born	28.6	(27.5–29.7)	14.3	(13.7–15.0)	12.6	(11.4–13.9)	50.7	(45.8–55.5)	70.5	(64.3–76.1)	81.1	(78.1–83.7)
	Foreign-born	59.7	(58.0–61.4)	16.2	(15.5–16.9)	12.9	(11.9–14.0)	33.6	(29.6–37.9)	63.5	(57.4–69.2)	72.9	(69.7–83.7)
**Puerto Rican**	Overall	20.7	(19.1–22.5)	15.9	(14.7–17.1)	15.1	(13.2–17.2)	57.5	(50.5–64.3)				
	Male	24.2	(21.8–26.7)	16.0	(14.4–17.8)	13.0	(10.4–16.1)	53.6	(42.6–64.3)				
	Female	17.5	(15.6–19.7)	15.8	(14.3–17.4)	17.0	(14.4–19.8)	61.1	(52.8–68.9)	71.7	(63.8–78.4)	83.8	(79.7–87.3)
	U.S.-born	20.2	(18.6–22.0)	15.9	(14.7–17.2)	15.1	(13.2–17.3)	57.9	(50.8–64.7)	72.0	(64.0–78.8)	83.5	(79.2–87.0)
	Foreign-born	38.4	(26.6–51.8)	17.0	(11.2–25.0)	—¶¶¶	—¶¶¶	—¶¶¶	—¶¶¶	—¶¶¶	—¶¶¶	96.7	(78.0–99.6)
**Cuban**	Overall	32.1	(28.7–35.7)	16.3	(14.5–18.4)	9.0	(7.2–11.3)	40.0	(32.1–48.5)				
	Male	35.8	(31.9–39.9)	14.2	(12.3–16.5)	8.0	(5.9–10.8)	29.1	(18.8–42.0)				
	Female	28.0	(24.0–32.4)	18.7	(16.2–21.5)	10.3	(7.4–14.2)	49.0	(37.0–61.1)	61.2	(50.1–71.2)	76.8	(69.2–83.0)
	U.S.-born	15.7	(12.3–19.8)	15.8	(12.3–20.0)	7.6	(4.9–11.6)	68.5	(38.5–88.3)	—¶¶¶	—¶¶¶	89.5	(77.4–95.5)
	Foreign-born	41.2	(36.8–45.9)	17.7	(15.4–20.3)	9.5	(7.2–12.5)	37.6	(29.4–46.6)	61.3	(50.0–71.5)	71.6	(62.3–79.4)
**Central American or South American**	Overall	45.8	(43.8–47.9)	15.6	(14.8–16.6)	11.5	(10.3–12.9)	41.4	(36.1–47.0)				
	Male	50.9	(48.5–53.3)	14.6	(13.5–15.8)	9.8	(8.3–11.6)	41.4	(33.7–49.6)				
	Female	40.5	(38.2–42.7)	16.7	(15.6–17.9)	13.3	(11.4–15.5)	41.4	(34.3–48.9)	69.3	(61.7–76.0)	77.7	(73.9–81.2)
	U.S.-born	25.6	(22.8–28.6)	15.5	(12.6–19.0)	9.1	(6.3–13.0)	84.3	(48.8–96.8)	—¶¶¶	—¶¶¶	76.3	(63.2–85.9)
	Foreign-born	50.3	(48.1–52.5)	15.9	(15.0–17.0)	11.8	(10.4–13.2)	40.1	(34.7–45.7)	69.4	(61.7–76.2)	78.1	(74.0–81.7)

**Abbreviation:** CI = confidence interval.

* All data are from NHIS, pooled for 2011–2013 for uninsured prevalences, 2009–2013 for delay or nonreceipt of medical care or prescription drugs because of cost, and pooled for 2010 and 2013 for colorectal testing, mammography, and Papanicolau (Pap) tests. Calculations based on ages 18–64 years for health insurance and nonutilization because of cost, 50–75 years for colorectal testing, 50–74 years for mammography, and 21–65 years for Pap tests. Estimates are based on household interviews of a sample of the civilian noninstitutionalized adult population. Unknowns for the columns were not included in the denominators when calculating percentages. Percentages might not add to totals because of rounding. “All adults” includes other races not shown separately.

† Uninsured defined as not having any private health insurance, Medicare, Medicaid, Children’s Health Insurance Program (CHIP), state-sponsored or other government-sponsored health plan, or military plan, or having only Indian Health Service coverage or only a private plan that paid for one type of service, such as accidents or dental care.

§ Delay or nonreceipt of needed medical care during the past 12 months because of cost was based on response to the questions, “During the past 12 months was there any time when person needed medical care but did not get it because person couldn’t afford it?” and “During the past 12 months has medical care been delayed because of worry about the cost?”

¶ Age-adjusted to the 2000 U.S. standard population for ages 18–64 using age groups 18–44, 45–54, and 55–64 years.

** Nonreceipt of needed prescription drugs during the past 12 months because of cost was based on response to the question, “During the past 12 months was there any time when person needed prescription medicine but didn’t get it because person couldn’t afford it?”

†† Use of colorectal tests or procedures includes reports of home fecal occult blood test (FOBT) in the past year, sigmoidoscopy procedure in the past 5 years with FOBT in the past 3 years, or colonoscopy procedure in the past 10 years. In 2008, the U.S. Preventive Services Task Force recommended screening for colorectal cancer annually using FOBT, every 5 years using sigmoidoscopy with FOBT every 3 years, or every 10 years using colonoscopy, in adults beginning at age 50 years and continuing until age 75 years. Additional information available at http://www.uspreventiveservicestaskforce.org/uspstf08/colocancer/colors.htm.

§§ Use of mammography was based on the following: female respondents aged ≥40 years were asked “Have you ever had a mammogram?” Those who responded “yes” were then asked about the date and time of their most recent mammogram. The U.S. Preventive Services Task Force recommends biennial screening mammography for women aged 50–74 years; however, some persons might start earlier screening because of higher associated risks. The table presents crude estimates for women aged 50–74 years who received a mammogram in the past 2 years.

¶¶ Use of Pap tests based on the following: in NHIS, female respondents aged ≥18 years were asked, “Have you ever had a Pap smear or Pap test?” Those who responded “yes” were then asked about the date and time of their most recent Pap test. Using recommendations of the U.S. Preventive Services Task Force, the table presents crude estimates for women aged 21–65 years without a hysterectomy who received a Pap test in the past 3 years.

*** “U.S.-born” refers to persons born in the 50 states, District of Columbia, or U.S territories and includes children born outside the United States to U.S. citizens.

††† “Foreign-born” refers to persons born outside the United States or its territories (except for children of U.S. citizens), regardless of current citizenship.

§§§ Persons of Hispanic/Latino ethnicity can be of any race or combination of races.

¶¶¶ Estimate has a relative standard error >30%.

## Footnotes

* According to the U.S. Office of Management and Budget, “Hispanic or Latino” refers to a “person of Cuban, Mexican, Puerto Rican, South or Central American, or other Spanish culture or origin regardless of race. Hispanic origin can be viewed as the heritage, nationality group, lineage, or country of birth of the person or the person’s parents or ancestors before their arrival in the United States.”

† Hispanic origin here refers to self-reported Hispanic ethnic/cultural heritage, regardless of race(s) or place of birth. For example, in this analysis, “Mexicans” refers to persons who trace their cultural roots to Mexico, but who were not necessarily born in Mexico.

§ The definitions of “U.S.-born” and “foreign-born” differ slightly for the two major national health surveys used in this report (the National Health Interview Survey [NHIS] and the National Health and Nutrition Examination Survey [NHANES]). In NHIS, “U.S. born” refers to persons born in the 50 states, District of Columbia or U.S territories and includes children born outside the United States to U.S parents. In NHANES, “U.S.-born” refers to persons born in the 50 states or District of Columbia. In NHIS, “foreign born” refers to persons born outside the United States or its territories (except children of U.S. citizens), regardless of current citizenship. In NHANES, “foreign-born” refers to all persons born outside the United States, regardless of current citizenship.

¶ The American Community Survey is “an ongoing survey conducted by the U.S. Census Bureau” that “uses a series of monthly samples to produce annually updated estimates for the census tracts and block groups formerly surveyed via the decennial census long-form sample.” This provides “communities the current information they need to plan investments and services.” Additional information available at http://www.census.gov/acs/www.

** NHIS is a survey of a representative sample of the civilian, noninstitutionalized U.S. household population. Only data from Hispanic origin groups having estimates with a relative standard error ≤30% are reported.

†† NHANES is “a program of studies designed to assess the health and nutritional status of adults and children in the United States.” “The survey is unique in that it combines interviews and physical examinations. The sample for the survey is selected to represent the U.S. population of all ages.” Additional information available at http://www.cdc.gov/nchs/nhanes/about_nhanes.htm.
